# Bacterial incidence and drug resistance from pathogens recovered from blood, cerebrospinal and pleural fluids in 2019–2020. Results of the Invifar network

**DOI:** 10.7717/peerj.14411

**Published:** 2023-01-17

**Authors:** Elvira Garza-González, Adrian Camacho-Ortiz, Alfredo Ponce-de-Leon, Edgar Ortiz-Brizuela, Luis Esaú López-Jácome, Claudia Colin, Fabian Rojas-Larios, Oscar A. Newton-Sánchez, Gabriela Echaniz-Aviles, Maria Noemi Carnalla-Barajas, Araceli Soto, Paola Bocanegra-Ibarias, Ana María del Rocío Hernández-Dueñas, María del Consuelo Velázquez-Acosta, Laura Karina Avilés-Benítez, Juan Pablo Mena-Ramirez, Daniel Romero, Isela Mora-Jiménez, Margarita Alcaraz-Espejel, José Manuel Feliciano-Guzmán, Maribel López-García, Patricia Rodriguez-Zulueta, María Angelina Quevedo-Ramos, Cecilia Padilla-Ibarra, Carlos Antonio Couoh-May, Maria Carolina Rivera-Ferreira, Cecilia Teresita Morales-de-la-Peña, Hector Zubiate, Raúl Peralta-Catalán, Carlos Miguel Cetina-Umaña, Joaquin Rincón-Zuno, Maria Lucia Perez-Ricardez, Iris Yazmin Hernández-Cordova, Eduardo López-Gutiérrez, Mariana Gil, Efren Aguirre-Burciaga, Guadalupe Soledad Huirache-Villalobos, Scarlett Munoz, Nicolás Rogelio Eric Barlandas-Rendón, Enrique Bolado-Martinez, Luis Javier Quintanilla-Cazares, Abraham C. Gómez-Choel, Laura Lopez, Juan Carlos Tinoco, Rosa Areli Martínez-Gamboa, Alejandro Molina, Samuel Pavel Escalante-Armenta, Lizbeth Duarte, Luis Alberto Ruiz-Gamboa, Dulce Isabel Cobos-Canul, Dulce López, Irma Elena Barroso-Herrera-y-Cairo, Eduardo Rodriguez-Noriega, Rayo Morfin-Otero

**Affiliations:** 1 Facultad de Medicina, Hospital Universitario Dr. José Eleuterio González, Universidad Autónoma de Nuevo León, Monterrey, Nuevo León, Mexico; 2 Instituto Nacional de Ciencias Médicas y Nutrición Salvador Zubirán, Mexico City, Mexico; 3 Instituto Nacional de Rehabilitación Luis Guillermo Ibarra Ibarra, Mexico City, Mexico; 4 Facultad de Medicina, Universidad de Colima and Hospital Regional Universitario de los Servicios de Salud del Estado de Colima, Colima, Mexico; 5 Instituto Nacional de Salud Pública, Cuernavaca, Mexico; 6 Instituto Nacional de Cardiología Ignacio Chávez, Mexico City, Mexico; 7 Instituto Nacional de Cancerología, Mexico City, Mexico; 8 Hospital infantil de Morelia “Eva Sámano de López Mateos”, Morelia, Mexico; 9 Hospital General de Zona No.21 IMSS, Centro Universitario de los Altos (CUALTOS), Universidad de Guadalajara, Guadalajara, Mexico; 10 Análisis Bioquímico Clínicos “Louis Pasteur”, Toluca, Mexico; 11 Centenario Hospital Miguel Hidalgo, Aguascalientes, Mexico; 12 Sanatorio la Luz, Morelia, Mexico; 13 Hospital de Especialidades Pediátricas, Tuxtla Gutiérrez, Mexico; 14 Hospital de la Madre y el Niño Guerrerense, Chilpancingo, Mexico; 15 Hospital Dr. Manuel GEA González, Mexico City, Mexico; 16 Hospital General de León, León, México; 17 Hospital General del Estado Doctor Ernesto Ramos Bours, Hermosillo, Mexico; 18 Hospital General Dr Agustin O’Horán, Mérida, Mexico; 19 Hospital General Dr. Miguel Silva, Morelia, Mexico; 20 Hospital General Juan María de Salvatierra, La Paz, Mexico; 21 Hospital General Lázaro Cárdenas, ISSSTE, Chihuahua, Mexico; 22 Hospital General Raymundo Abarca Alarcón, Chilpancingo, Mexico; 23 Hospital Materno Infantil Morelos, Chetumal, Mexico; 24 Hospital para el Niño de Toluca, IMIEM, Toluca, Mexico; 25 Hospital para el Niño Poblano, Puebla, Mexico; 26 Hospital Regional de Alta Especialidad Bicentenario de la Independencia, Tultitlán de Mariano Escobedo, Mexico; 27 Hospital Regional de Alta Especialidad de Oaxaca, Oaxaca, Mexico; 28 Hospital Regional de Alta Especialidad del Bajío, Leon, Mexico; 29 Hospital Regional de Delicias, Delicias, Mexico; 30 Laboratorios del Centro, Zamora, Mexico; 31 Swisshospital, Monterrey, Mexico; 32 Universidad Autónoma de Guerrero and Laboratorio Bioclin, Chilpancingo, Mexico; 33 Universidad de Sonora, Hermosillo, Mexico; 34 Hospital Ángeles Valle Oriente, San Pedro Garza García, Mexico; 35 Hospital General De Zona No.1 IMSS Nueva Frontera, Tapachula, Mexico; 36 Hospital Galenia, Cancún, Mexico; 37 Hospital general de Durango, Durango, Mexico; 38 Centro Médico Dr. Ignacio Chávez, Hermosillo, Mexico; 39 Hospital General de Ciudad Obregón, Ciudad Obregón, Mexico; 40 Centro Integral de Atención a la Salud Sur ISSSTESON, Hermosillo, Mexico; 41 Hospital Dr. Jesús Gilberto Gómez Maza, Tuxtla Gutiérrez, Mexico; 42 Hospital General Chetumal, Chetumal, Mexico; 43 Hospital Lic. Adolfo López Mateos, Ciudad Obregón, Mexico; 44 Hospital “Dr. Fernando Ocarranza”, Hermosillo, Mexico; 45 Hospital Civil de Guadalajara Fray Antonio Alcalde, Universidad de Guadalajara, Guadalajara, Jalisco, Mexico; 46 Hospital Civil de Guadalajara Fray Antonio Alcalde, Universidad de Guadalajara, Guadalajara, Jalisco, Mexico

**Keywords:** Drug resistance, Blood stream infection, Cerebrospinal infection, *Escherichia coli*, INVIFAR

## Abstract

**Background:**

Antimicrobial resistance is a global concern. Analysis of sterile fluids is essential because microorganisms are defined as significant in most cases. Blood, cerebrospinal, and pleural fluids are frequently received in the microbiology lab because they are associated with considerable rates of morbi-mortality. Knowledge of epidemiology in these samples is needed to choose proper empirical treatments due to the importance of reducing selection pressure.

**Methods:**

We used retrospective laboratory data of blood, CSF, and pleural fluid collected from patients in Mexico between 2019 and 2020. Each laboratory identified the strains and tested susceptibility using its routine methods. For *Streptococcus pneumoniae*, a comparative analysis was performed with data from the broth microdilution method.

**Results:**

Forty-five centers participated in the study, with 30,746 clinical isolates from blood, 2,429 from pleural fluid, and 2,275 from CSF. For blood and CSF, *Staphylococcus epidermidis* was the most frequent. For blood, among gram negatives, the most frequent was *Escherichia coli*. Among *Enterobacterales*, 9.8% of *K. pneumoniae* were carbapenem-resistant*.* For *S. pneumoniae*, similar resistance percentages were observed for levofloxacin, cefotaxime, and vancomycin. For CSF, the most frequent gram-negative was *E. coli.* In *Acinetobacter baumannii*, carbapenem resistance was 71.4%. The most frequent species detected for pleural fluid was *E. coli*; in *A. baumannii*, carbapenem resistance was 96.3%.

**Conclusion:**

Gram-negative bacteria, with *E. coli* most prevalent, are frequently recovered from CSF, blood, and pleural fluid. In *S. pneumoniae*, the routine, conventional methods showed good agreement in detecting resistance percentages for erythromycin, levofloxacin, and vancomycin.

## Introduction

Bacterial species are developing resistance to many antibiotics (Prestinaci, Pezzotti & Pantosti, 2015; Paphitou, 2013; Karam & Heffner, 2000; Laxminarayan et al., 2013; Pogue et al., 2015). To control the dissemination of these organisms, it is necessary to monitor the antimicrobial susceptibility of the microorganisms most frequently detected in hospitals (Roca et al., 2015). The analysis of sterile fluids is essential because potential pathogens are considered significant in most cases (Opota et al., 2015). Blood, cerebrospinal fluid (CSF), and pleural fluid are sterile fluids frequently received in the microbiology laboratory for culture in suspected infections. These infections are associated with considerable morbidity and mortality (Proulx et al., 2005).

Regarding blood cultures, mortality associated with bloodstream infections is directly related to the delay in administering the first adequate antimicrobial agent (Ferrer et al., 2014). Empirical antimicrobial infectious treatments are chosen based on clinical and epidemiological data (Paul et al., 2010).

Bacterial meningitis occurs primarily in childhood and is associated with a high mortality rate and potentially severe morbidity. Antibiotic resistance may limit the effectiveness of treatment (Kim, 2010; Briand et al., 2016). Thus, early diagnosis and appropriate antibiotic therapy are needed to avoid further complications.

Pleural infections are a significant cause of morbidity or death, and their incidence continues to rise (Farjah et al., 2007). Current guidelines recommend pleural fluid microbiological studies to direct antibiotic treatment (Light, 1999). Mortality has been found to increase as much as 40% when gram-negative bacteria, *Staphylococcus aureus,* or a mixed aerobic infection are detected (Maskell et al., 2006). Thus, reliable identification of these groups would allow the targeting of early aggressive therapy, and calculations of resistance percentages may help in the use of adequate empirical treatments.

It is necessary to define local and national resistance rates for various pathogens in the blood and other sterile body fluids to provide baseline data that can serve as an essential reference for monitoring resistance and empirical therapy changes. Thus, the present study investigated the cumulative incidence and antimicrobial susceptibility patterns from pathogens recovered from sterile fluids.

## Material and Methods

### Ethics statement

This study was performed in compliance with the requirements of the Research and Biosafety Ethics Committee of the Antiguo Hospital Civil de Guadalajara “Fray Antonio Alcalde,” Jalisco, Mexico, which approved this study with reference number 129/17. The ethics committee waived informed consent because no intervention was involved, and no patient-identifying information was included. All institutions agreed to participate.

### Participating centers, data collection, and analysis

The centers from the Network for the Research and Surveillance of Drug Resistance (Red Temática de Investigación y Vigilancia de la Farmacorresistencia, INVIFAR in Spanish) were invited to participate in the study. The INVIFAR network was created in March 2018 to contribute to improving the surveillance and control of drug resistance in Mexico and the performance of the network’s laboratories, comprised of laboratories, hospitals, research centers, and universities in Mexico. Participating centers provided retrospective aggregated laboratory data of blood, CSF, and pleural liquid collected from January 1, 2019, to December 31, 2020.

Each laboratory identified the strains and tested their susceptibilities using routine, conventional methods, including commercial microdilution systems (VITEK 2, Biomérieux; Phoenix Automated Microbiology System, Becton, Dickinson; MicroScan WalkAway, Siemens Healthcare Diagnostics; and Sensititre, TREK Diagnostic Systems Inc.) or the disk diffusion susceptibility method.

All databases were deposited into the WHONET 5.6 platform and converted to the WHONET format using the BacLink 2 tool. Only one strain per patient was included. The distribution of antimicrobial resistance was included for clinical isolates recovered from blood, CSF, and pleural liquid. Clinical and Laboratory Standards Institute (CLSI) criteria were used to score the results (CLSI, 2020). Databases included type of body fluid, isolated bacterial species, antibiotic tested, and susceptibility test results.

### Susceptibility testing for *Streptococcus pneumoniae*

For *S. pneumoniae*, a comparative analysis was conducted between results observed for routine, conventional methods with data from the broth microdilution (BMD) method. Only blood isolates were included in this study. This work was performed with the collaboration of the SIREVA/GIVEBPVac (Grupo Interinstitutional para la Vigilancia de Enfermedades Bacterianas Prevenibles por Vacunación, in Spanish) network. Susceptibility testing was performed by BMD following the guidelines of the CLSI (2020). *S. pneumoniae* ATCC 49619 was used as a control strain. Cation-adjusted Mueller Hinton broth (Becton, Dickinson, MD, USA), which had lysed horse blood added to a final concentration of 5%, was used as a medium.

## Results

### Participating centers and included clinical isolates

Forty-five centers participated in the study, contributing 35,450 clinical isolates recovered from sterile fluids, which included 30,746 from blood, 2,429 from pleural fluid, and 2,275 from CSF.

The most frequently detected bacterial species in blood and CSF was *Staphylococcus epidermidis*. After *S. epidermidis* in blood samples, gram-negative isolates were frequently detected: *Escherichia coli* was the most frequent species, followed by *Klebsiella pneumoniae* and *Enterobacter cloacae*. Other frequently detected species were *Serratia marcescens*, *Klebsiella aerogenes*, *Proteus mirabilis*, *Morganella morganii*, and *Salmonella* spp*.* Among non-fermenters, the most frequent species were *Pseudomonas aeruginosa*, followed by *Acinetobacter baumannii*, *Stenotrophomonas maltophilia*, and *Burkholderia cepacia*. Among gram-positive isolates, *S. epidermidis* was the most frequent species detected, followed by *S. aureus.*

In clinical isolates from CSF, gram-negative bacteria were frequently detected, with *E. coli* being the most prevalent, followed by *K. pneumoniae* and non-fermenters *P. aeruginosa* and *A. baumannii*. Again, *S. epidermidis* was the most frequent species detected among gram-positive bacteria, followed by *S. aureus*.

In clinical isolates from pleural fluid, the most frequent species detected among gram-negative bacteria was *E. coli,* followed by *P. aeruginosa*. Other species detected were *K. pneumoniae*, *E. cloacae*, *P. mirabilis*, and *A. baumannii.* In gram-positive isolates, *S. aureus* was the most frequently detected species.

### Drug resistance of pathogens from blood

For pathogens recovered from blood, among Enterobacterales (except *Salmonella* species), carbapenem resistance ranged from 0% in *K. aerogenes* to 9.8% in *K. pneumoniae.* Quinolone resistance ranged from 0% to 35.8%, with higher percentages detected for ciprofloxacin than for levofloxacin, except in *C. freundii* and *S. marcescens* (Table 1A). The highest resistance was observed in *E. coli* (64% for ciprofloxacin and 57.8% for levofloxacin). In general, high resistance percentages were observed for cephalosporins, with lower values observed in *E. cloacae*.

**Table 1:** Distribution of resistant (R), intermediate (I), and susceptible (S) gram-negative isolates from blood samples of patients in Mexico.

	*E. coli*				*K. pneumoniae*				*K. aerogenes*				*E. cloacae*				*C. freundii*				*S. marcescens*				*P. mirabilis*				*M. morganii*			
**Antibiotic**	**n**	**%R**	**%I**	**%S**	**n**	**%R**	**%I**	**%S**	**n**	**%R**	**%I**	**%S**	**n**	**%R**	**%I**	**%S**	**n**	**%R**	**%I**	**%S**	**n**	**%R**	**%I**	**%S**	**n**	**%R**	**%I**	**%S**	n	%R	%I	%S
AMP	1602	87.2	0.2	12.6	ND	ND	ND	ND	ND	ND	ND	ND	ND	ND	ND	ND	ND	ND	ND	ND	ND	ND	ND	ND	52	44.2	0.0	55.8	ND	ND	ND	ND
FOX	660	40.3	1.1	58.6	403	50.9	1	48.1	ND	ND	ND	ND	ND	ND	ND	ND	ND	ND	ND	ND	ND	ND	ND	ND	16	0.0	0.0	100	ND	ND	ND	ND
CXM	884	68.3	0.7	31	641	69.1	0.5	30.4	ND	ND	ND	ND	ND	ND	ND	ND	ND	ND	ND	ND	ND	ND	ND	ND	ND	ND	ND	ND	ND	ND	ND	ND
CTX	655	74.2	0.2	25.6	557	71.1	0.0	28.9	ND	ND	ND	ND	ND	ND	ND	ND	ND	ND	ND	ND	ND	ND	ND	ND	32	43.8	0.0	56.2	ND	ND	ND	ND
CAZ	1266	62.6	1.6	35.8	907	67.4	0.7	31.9	ND	ND	ND	ND	ND	ND	ND	ND	ND	ND	ND	ND	ND	ND	ND	ND	48	27.1	2.1	70.8	ND	ND	ND	ND
FEP	2263	56	2.4	41.6	1380	58.5	2.8	38.7	80	6.2	0.0	93.8	509	5.7	3.7	90.6	55	13	0.0	87	217	2.8	3.7	93.5	79	25.3	1.3	73.4	22	0.0	0.0	100
ATM	389	58.4	0.0	41.6	225	58.2	0.0	41.8	15	0.0	0.0	100	84	23.8	0.0	76.2	ND	ND	ND	ND	27	3.7	0	96.3	ND	ND	ND	ND	ND	ND	ND	ND
MEM	2320	3.5	0.2	96.3	1382	9.8	0.4	89.8	80	0.0	0.0	100	501	3.4	0.2	96.4	54	3.7	0.0	96	210	5.7	0.5	93.8	81	1.2	0	98.8	ND	ND	ND	ND
IPM	1587	3	0.7	96.3	816	12	0.1	87.9	52	0.0	7.7	92.3	293	4.1	4.1	91.8	35	2.9	11	86	46	8.7	8.7	82.6	23	56.5	34.8	8.7	10	10	0	90
SAM	1636	49.9	23.2	26.9	1092	62	4.2	33.8	ND	ND	ND	ND	ND	ND	ND	ND	ND	ND	ND	ND	ND	ND	ND	ND	58	19	0	81	ND	ND	ND	ND
AMC	395	27.1	13.4	59.5	203	34	14.8	51.2	ND	ND	ND	ND	ND	ND	ND	ND	ND	ND	ND	ND	ND	ND	ND	ND	ND	ND	ND	ND	ND	ND	ND	ND
TZP	1722	12.4	5.7	81.9	906	20.1	11.4	68.5	63	30.2	6.3	63.5	329	14.6	3.3	82.1	39	5.1	5.1	90	44	6.8	6.8	86.4	47	0.0	2.1	97.9	19	0.0	0.0	100
AMK	1187	3.5	0.3	96.2	965	8.1	1.1	90.8	53	1.9	0.0	98.1	367	4.1	0.3	95.6	39	5.1	0.0	95	151	9.9	0.7	89.4	46	2.2	4.3	93.5	ND	ND	ND	ND
GEN	2346	39.9	1.2	58.9	1406	45.5	2.3	52.2	80	5	0.0	95	512	7.8	0.2	92	56	14	0.0	86	217	1.4	0.0	98.6	82	13.4	19.5	67.1	23	21.7	13	65.2
TOB	198	39.4	15.7	44.9	199	54.8	8	37.2	14	0.0	0.0	100	58	12.1	1.7	86.2	ND	ND	ND	ND	20	0.0	0.0	100	ND	ND	ND	ND	ND	ND	ND	ND
CIP	2358	64	2.2	33.8	1395	25.3	17.1	57.6	80	3.8	2.5	93.7	510	5.1	2	92.9	55	9.1	3.6	87	217	1.4	0.9	97.7	81	35.8	3.7	60.5	23	30.4	13	56.5
LVX	735	57.8	0.8	41.4	347	11.2	2.3	86.5	19	0.0	0.0	100	134	3.7	1.5	94.8	20	15	0.0	85	41	2.4	4.9	92.7	12	16.7	16.7	66.6	ND	ND	ND	ND
SXT	1596	62.3	0.1	37.6	979	59.8	0.0	40.2	47	4.3	0.0	95.7	361	15.5	0.0	84.5	41	27	0.0	73	169	8.9	0.0	91.1	54	40.7	0.0	59.3	14	57.1	0.0	42.9

	*Salmonella* spp.				*S.* Typhi				*A. baumannii*				*P. aeruginosa*				*S. maltophilia*				*B. cepacia*			
Antibiotic	n	%R	%I	%S	n	%R	%I	%S	n	%R	%I	%S	n	%R	%I	%S	n	%R	%I	%S	n	%R	%I	%S
AMP	70	32.9	0.0	67.1	65	0	0	100	ND	ND	ND	ND	ND	ND	ND	ND	ND	ND	ND	ND	ND	ND	ND	ND
FOX	ND	ND	ND	ND	ND	ND	ND	ND	ND	ND	ND	ND	ND	ND	ND	ND	ND	ND	ND	ND	ND	ND	ND	ND
CXM	ND	ND	ND	ND	ND	ND	ND	ND	ND	ND	ND	ND	ND	ND	ND	ND	ND	ND	ND	ND	ND	ND	ND	ND
CTX	23	4.3	0.0	95.7	63	0.0	0.0	100	ND	ND	ND	ND	ND	ND	ND	ND	ND	ND	ND	ND	ND	ND	ND	ND
CAZ	69	1.4	0.0	98.6	69	0.0	0.0	100	557	74.7	3.9	21.4	ND	ND	ND	ND	ND	ND	ND	ND	42	0.0	11.9	88.1
FEP	64	0.0	4.7	95.3	66	0.0	0.0	100	642	71.8	0.9	27.3	906	21.3	5.7	73	ND	ND	ND	ND	ND	ND	ND	ND
ATM	ND	ND	ND	ND	ND	ND	ND	ND	ND	ND	ND	ND	109	21.1	2.8	76.1	ND	ND	ND	ND	ND	ND	ND	ND
MEM	ND	ND	ND	ND	ND	ND	ND	ND	729	72.6	0.4	27	893	30	5.4	64.6	ND	ND	ND	ND	60	5.0	1.7	93.3
IPM	ND	ND	ND	ND	ND	ND	ND	ND	386	79.5	0.3	20.2	590	36.1	1.7	62.2	ND	ND	ND	ND	ND	ND	ND	ND
SAM	ND	ND	ND	ND	ND	ND	ND	ND	663	59.9	13.6	26.5	ND	ND	ND	ND	ND	ND	ND	ND	ND	ND	ND	ND
TZP	ND	ND	ND	ND	ND	ND	ND	ND	455	84.8	0.4	14.8	642	22.6	9	68.4	ND	ND	ND	ND	ND	ND	ND	ND
AMK	ND	ND	ND	ND	ND	ND	ND	ND	125	58.4	9.6	32	576	13.7	3.5	82.8	ND	ND	ND	ND	ND	ND	ND	ND
GEN	ND	ND	ND	ND	ND	ND	ND	ND	736	55.8	11.3	32.9	900	15.1	5	79.9	ND	ND	ND	ND	ND	ND	ND	ND
TOB	ND	ND	ND	ND	ND	ND	ND	ND	118	69.5	11	19.5	80	25	0.0	75	ND	ND	ND	ND	ND	ND	ND	ND
CIP	88	11.4	9.1	31.8	65	11	0	91	730	73.4	0.1	26.5	ND	ND	ND	ND	ND	ND	ND	ND	ND	ND	ND	ND
LVX	ND	ND	ND	ND	ND	ND	ND	ND	146	62.3	1.4	36.3	ND	ND	ND	ND	110	3.6	6.4	90	ND	ND	ND	ND
SXT	70	25.7	0.0	74.3	65	0.0	0.0	100	441	63.3	0.0	36.7	ND	ND	ND	ND	313	9.3	0.3	90.4	39	20.5	0.0	79.5

**Notes.**

AMP ampicillin FOX cefoxitin CXM cefuroxime CTX cefotaxime CAZ ceftazidime FEP cefepime ATM aztreonam MEM meropenem IPM imipenem SAM ampicillin/sulbactam TZP piperacillin/tazobactam AMK amikacin GEN gentamicin TOB tobramycin CIP ciprofloxacin LVX levofloxacin SXT trimethoprim-sulfamethoxazole ND not determined

A high susceptibility was observed for *Salmonella enterica* serovar Typhi (*S.* Typhi) strains, with resistance detected for ciprofloxacin (11%). Concerning non-fermenters, more than 60% of *A. baumannii* were resistant to most antibiotics tested; the highest resistance observed in *P. aeruginosa* was for imipenem (36.1%). In *B. cepacia,* the lowest resistance rate was detected for ceftazidime (0%) (Table 1B).

Among gram-positive isolates, almost 30% of *S. aureus* strains were methicillin-resistant, and 19% of *E. faecium* were vancomycin-resistant. Results from more than 50 strains were obtained for *S. pneumoniae*, with the highest percentage of resistance detected for trimethoprim-sulfamethoxazole (42.1%; Table 2). Compared with the BMD method, similar resistance percentages were observed for levofloxacin, erythromycin, and vancomycin. However, values observed for chloramphenicol and trimethoprim/sulfamethoxazole were higher using the BMD method than routine, conventional methods. There was a discrepancy in resistance to penicillin and cefotaxime that could not be explained; perhaps it is associated with a limited number of strains with conventional routine methods.

**Table 2:** Distribution of resistant (R), intermediate (I), and susceptible (S) gram-positive isolates from blood samples of patients in Mexico.

	*S. aureus*				*S. epidermidis*				*E. faecalis*				*E. faecium*				*S. pneumoniae* ^a^				*S. pneumoniae* ^b^				*Streptococcus* spp. v*iridans* group			
Antibiotic	n	%R	%I	%S	n	%R	%I	%S	n	%R	%I	%S	n	%R	%I	%S	n	%R	%I	%S	n	%R	%I	%S	n	%R	%I	%S
PEN	353	90.4	0.0	9.6	576	94.8	0.0	5.2	127	15.7	0.0	84.3	55	78.2	0.0	21.8	13	23.1	30.8	46.2	49	12.2	14.3	73.5	26	3.8	7.7	88.5
AMP	ND	ND	ND	ND	ND	ND	ND	ND	589	1.5	0.0	98.5	247	64	0.0	36	ND	ND	ND	ND	ND	ND	ND	ND	25	8	0.0	92
OXA	1250	27.1	0.0	72.9	2899	82.7	0.0	17.3	ND	ND	ND	ND	ND	ND	ND	ND	ND	ND	ND	ND	ND	ND	ND	ND	ND	ND	ND	ND
FOX	656	29.9	0.0	70.1	1980	85.5	0.0	14.5	ND	ND	ND	ND	ND	ND	ND	ND	ND	ND	ND	ND	ND	ND	ND	ND	ND	ND	ND	ND
CTX	ND	ND	ND	ND	ND	ND	ND	ND	ND	ND	ND	ND	ND	ND	ND	ND	27	14.8	3.7	81.5	49	16.3	20.4	63.3	ND	ND	ND	ND
FEP	ND	ND	ND	ND	ND	ND	ND	ND	ND	ND	ND	ND	ND	ND	ND	ND	ND	ND	ND	ND	ND	ND	ND	ND	26	3.8	0	96.2
CIP	1222	26.8	1.1	72.1	2910	61.8	4.4	33.8	526	31.9	4	64.1	220	47.7	20.5	31.8	ND	ND	ND	ND	ND	ND	ND	ND	ND	ND	ND	ND
LVX	903	25.9	0.7	73.4	2399	67.5	0.5	32	461	28.9	0.4	70.7	187	44.4	11.2	44.4	58	1.7	1.7	96.6	49	2.0	2.0	96.0	ND	ND	ND	ND
ERY	1260	32.1	0.7	67.2	2852	80.8	0.6	18.7	539	59	28.4	12.6	220	76.8	19.5	3.6	55	34.5	0	65.5	49	44.9	0.0	55.1	ND	ND	ND	ND
CLI	1230	31.3	0.2	68.5	2790	72.1	0.7	27.2	ND	ND	ND	ND	ND	ND	ND	ND	56	23.2	10.7	66.1	ND	ND	ND	ND	ND	ND	ND	ND
TET	977	3.2	0.1	96.7	2393	11.9	0.3	87.8	438	75.3	0	24.7	170	61.8	0	38.2	51	23.5	9.8	66.7	ND	ND	ND	ND	ND	ND	ND	ND
GEN	1224	8.9	3	88.1	2932	41.4	11.6	47	ND	ND	ND	ND	ND	ND	ND	ND	ND	ND	ND	ND	ND	ND	ND	ND	ND	ND	ND	ND
SXT	1286	4.1	0.0	95.9	2926	57.2	0.0	42.8	ND	ND	ND	ND	ND	ND	ND	ND	57	42.1	8.8	49.1	49	57.1	14.3	28.6	ND	ND	ND	ND
LZD	1255	0.2	0.0	99.8	2815	4.6	0.0	95.4	584	3.4	4.3	92.3	242	2.9	1.2	95.9	36	0.0	0.0	100	ND	ND	ND	ND	ND	ND	ND	ND
RIF	809	3.2	0.1	96.7	2211	10.8	0.0	1.4	ND	ND	ND	ND	ND	ND	ND	ND	ND	ND	ND	ND	ND	ND	ND	ND	ND	ND	ND	ND
CHL	ND	ND	ND	ND	ND	ND	ND	ND	ND	ND	ND	ND	ND	ND	ND	ND	29	6.9	0.0	93.1	49	36.7	0.0	63.3	ND	ND	ND	ND
VAN	1285	0.0	0.0	100	3002	0.0	0.0	100	588	2	0.0	98	247	19	0.0	81	58	0.0	0.0	100	49	0.0	0.0	100.0	26	0.0	0.0	100

**Notes.**

PEN penicillin AMP ampicillin OXA oxacillin FOX cefoxitin CTX cefotaxime FEP cefepime CIP ciprofloxacin LVX levofloxacin ERY erythromycin CLI clindamycin TET tetracycline GEN gentamicin SXT trimethoprim-sulfamethoxazole LZD linezolid RIF rifampicin CHL chloramphenicol VAN vancomycin ND not determined

a Data from isolates analyzed using the conventional routine methods.

b Data from isolates analyzed using the broth microdilution method.

### Drug resistance of pathogens from cerebrospinal fluid

For pathogens recovered from CSF, carbapenem resistance was as high as 71.4% in *A. baumannii*. Resistance rates higher than 44% for gentamicin and 61% for cephalosporins were detected in *E. coli* and *K. pneumoniae*. *E. coli* exhibited more than 60% resistance to quinolones. In *A. baumannii,* the resistance rates of most evaluated antibiotics were higher than 60% (Table 3). Among *E. coli* (*n* = 91), 86.9% showed to be extended-spectrum beta-lactamase producers; among *K. pneumoniae* (*n* = 39), 71.8%. Among gram-positive isolates, 44.4% of *S. aureus* showed resistance to methicillin, and 36.4% of *E. faecium* were resistant to vancomycin (Table 4).

**Table 3:** Distribution of resistant (R), intermediate (I), and susceptible (S) gram-negative isolates from the cerebrospinal fluid of patients in Mexico.

	*E. coli*				*K. pneumoniae*				*S. marcescens*				*E. cloacae*				*P. aeruginosa*				*A. baumannii*			
Antibiotic	n	%R	%I	%S	n	%R	%I	%S	n	%R	%I	%S	n	%R	%I	%S	n	%R	%I	%S	n	%R	%I	%S
AMP	112	94.6	0.0	5.4	ND	ND	ND	ND	ND	ND	ND	ND	ND	ND	ND	ND	ND	ND	ND	ND	ND	ND	ND	ND
FOX	24	66.7	0.0	33.3	14	21.4	7.1	71.5	ND	ND	ND	ND	ND	ND	ND	ND	ND	ND	ND	ND	ND	ND	ND	ND
CXM	63	81	0.0	19	50	80	0.0	20	ND	ND	ND	ND	ND	ND	ND	ND	ND	ND	ND	ND	ND	ND	ND	ND
CTX	60	78.3	0.0	21.7	37	75.7	0.0	24.3	ND	ND	ND	ND	ND	ND	ND	ND	ND	ND	ND	ND	17	88.2	0.0	11.8
CAZ	76	75	0.0	25	48	75	0.0	25	ND	ND	ND	ND	ND	ND	ND	ND	49	6.1	6.1	87.8	34	85.3	0.0	14.7
FEP	131	74	6.1	19.9	68	61.8	14.7	23.5	35	0.0	0.0	100	16	0	6.2	93.8	73	6.8	16.4	76.8	54	72.2	9.3	18.5
ATM	42	95.2	0.0	4.8	17	88.2	0.0	11.8	21	66.7	0.0	33.3	ND	ND	ND	ND	10	30	0	70	ND	ND	ND	ND
MEM	124	1.6	0.0	98.4	61	4.9	1.6	93.5	25	0.0	4	96	16	0	0	100	67	31.3	16.4	52.3	49	71.4	2	26.6
IPM	49	2	0.0	98	33	6.1	0.0	93.9	25	8	16	76	ND	ND	ND	ND	43	55.8	9.3	34.9	30	56.7	6.7	36.6
SAM	116	63.8	19.8	16.4	52	73.1	1.9	25	ND	ND	ND	ND	ND	ND	ND	ND	ND	ND	ND	ND	50	50	16	34
AMC	17	52.9	17.6	29.5	17	58.8	5.9	35.3	ND	ND	ND	ND	ND	ND	ND	ND	ND	ND	ND	ND	ND	ND	ND	ND
TZP	78	3.8	6.4	89.8	34	8.8	29.4	61.8	25	56	0.0	44	ND	ND	ND	ND	53	9.4	7.5	83.1	37	62.2	2.7	35.1
AMK	105	1.9	0.0	98.1	49	14.3	0.0	85.7	13	0.0	0.0	100	11	0.0	0.0	100	55	7.3	5.5	87.2	ND	ND	ND	ND
GEN	131	49.6	1.5	48.9	69	44.9	0.0	55.1	35	5.7	5.7	88.6	16	6.2	0.0	93.8	72	5.6	22.2	72.2	54	68.5	9.3	22.2
TOB	38	34.2	52.6	13.2	ND	ND	ND	ND	ND	ND	ND	ND	ND	ND	ND	ND	10	0.0	0.0	100	ND	ND	ND	ND
CIP	119	71.4	0.0	28.6	55	29.1	5.5	65.4	15	0.0	0.0	100	15	0.0	0.0	100	61	3.3	8.2	88.5	43	83.7	0	16.3
LVX	23	60.9	0.0	39.1	14	7.1	0.0	92.9	ND	ND	ND	ND	ND	ND	ND	ND	10	0	40	60	ND	ND	ND	ND
SXT	116	58.6	0.0	41.4	64	59.4	0.0	40.6	30	6.7	0.0	93.3	12	8.3	0.0	91.7	ND	ND	ND	ND	36	61.1	0.0	38.9

**Notes.**

AMP ampicillin FOX cefoxitin CXM cefuroxime CTX cefotaxime CAZ ceftazidime FEP cefepime ATM aztreonam MEM meropenem IPM imipenem SAM ampicillin/sulbactam AMC amoxicillin/clavulanate TZP piperacillin/tazobactam AMK amikacin GEN gentamicin TOB tobramycin CIP ciprofloxacin LVX levofloxacin SXT trimethoprim-sulfamethoxazole ND not determined

**Table 4:** Distribution of resistant (R), intermediate (I), and susceptible (S) gram-positive isolates from the cerebrospinal fluid of patients in Mexico.

	*S. aureus*				*S. epidermidis*				*E. faecium*				*E. faecalis*			
Antibiotic	n	%R	%I	%S	n	%R	%I	%S	n	%R	%I	%S	n	%R	%I	%S
PEN	19	73.7	0.0	26.3	ND	ND	ND	ND	ND	ND	ND	ND	ND	ND	ND	ND
AMP	16	81.2	0.0	18.8	ND	ND	ND	ND	11	63.6	0.0	36.4	22	0.0	0.0	100
OXA	68	35.3	0.0	64.7	224	75	0.0	25	ND	ND	ND	ND	ND	ND	ND	ND
FOX	45	44.4	0.0	55.6	139	77.7	0.0	22.3	ND	ND	ND	ND	ND	ND	ND	ND
CIP	59	32.2	3.4	64.4	219	43.8	8.2	48	10	60	10	30	15	26.7	6.7	66.6
LVX	49	34.7	0.0	65.3	187	50.3	0.0	49.7	ND	ND	ND	ND	15	26.7	0	73.3
ERY	60	43.3	0.0	56.7	208	70.2	1	28.8	10	90	0.0	10	15	33.3	46.7	20
CLI	59	47.5	0.0	52.5	208	53.4	1	45.6	ND	ND	ND	ND	ND	ND	ND	ND
TET	49	2	0.0	98	187	9.1	0.5	90.4	ND	ND	ND	ND	15	73.3	0.0	26.7
GEN	63	11.1	3.2	85.7	232	33.6	11.6	54.8	ND	ND	ND	ND	ND	ND	ND	ND
SXT	65	0	0.0	100	233	42.9	0.0	57.1	ND	ND	ND	ND	ND	ND	ND	ND
RIF	56	7.1	0.0	92.9	168	10.7	0.6	88.7	ND	ND	ND	ND	ND	ND	ND	ND
LZD	66	0.0	0.0	100	214	3.7	0.0	96.3	11	0.0	0.0	100	28	3.6	3.6	92.8
VAN	70	0.0	0.0	100	224	0.4	0.4	99.2	11	36.4	0.0	63.6	29	3.4	13.8	82.8

**Notes.**

PEN penicillin AMP ampicillin OXA oxacillin FOX cefoxitin CIP ciprofloxacin LVX levofloxacin ERY erythromycin CLI clindamycin TET tetracycline GEN gentamicin SXT trimethoprim-sulfamethoxazole RIF rifampicin LZD linezolid VAN vancomycin ND not determined

### Drug resistance of pathogens from pleural fluid

For pathogens recovered from pleural fluid, among gram-negative isolates, carbapenem resistance was as high as 96.3% in *A. baumannii*. Levofloxacin resistance was as high as 100% in *A. baumannii* and 87% in *E. coli* (Table 5). Resistance to third-generation cephalosporins was more than 68% in *E. coli* and *K. pneumoniae*. Moreover, 54% of *E. coli* (n = 85) and 60% of *K. pneumoniae* (n = 45) were shown to be extended-spectrum beta-lactamase producers. Among gram-positive isolates, methicillin resistance for *S. aureus* was almost 35%, and vancomycin resistance for *E. faecium* was 27.6% (Table 6).

**Table 5:** Distribution of resistant (R), intermediate (I), and susceptible (S) gram-negative isolates from pleural fluid of patients in Mexico.

	*E. coli*				*K. pneumoniae*				*E. cloacae*				*P. mirabilis*				*A. baumannii*				*P. aeruginosa*			
Antibiotic	n	%R	%I	%S	n	%R	%I	%S	n	%R	%I	%S	n	%R	%I	%S	n	%R	%I	%S	n	%R	%I	%S
AMP	94	86	1.1	12.8	ND	ND	ND	ND	ND	ND	ND	ND	ND	ND	ND	ND	ND	ND	ND	ND	ND	ND	ND	ND
FOX	35	40	2.9	57.1	10	20	0.0	80	ND	ND	ND	ND	ND	ND	ND	ND	ND	ND	ND	ND	ND	ND	ND	ND
CXM	67	76	0.0	23.9	34	82	0.0	72	ND	ND	ND	ND	10	60	0.0	40	ND	ND	ND	ND	ND	ND	ND	ND
CTX	66	76	0.0	24.2	33	82	0.0	41	ND	ND	ND	ND	10	60	0.0	40	27	33.3	18.5	48.2	ND	ND	ND	ND
CAZ	95	76	1.1	23.2	45	69	0.0	31	ND	ND	ND	ND	11	54.5	0.0	45.5	52	59.6	7.7	32.7	81	18.5	7.4	74.1
FEP	125	66	0.8	32.8	58	62	0.0	38	44	20.5	6.8	72.7	13	46.2	0.0	53.8	58	63.8	1.7	34.5	98	21.4	6.1	72.5
MEM	125	4	0.0	96	59	12	0.0	49	44	11.4	0.0	88.6	13	0.0	0.0	100	58	56.9	5.2	37.9	97	39.2	11.3	49.5
IPM	57	7	1.8	91.2	20	5	0.0	46	12	0	0.0	100	ND	ND	ND	ND	27	96.3	3.7	0.0	41	68.3	2.4	29.3
SAM	113	62	16	22.2	53	72	0.0	28	ND	ND	ND	ND	13	15.4	7.7	76.9	56	55.4	8.9	35.7	ND	ND	ND	ND
TZP	62	18	4.8	77.5	25	8	20	20	13	23.1	15.4	61.5	ND	ND	ND	ND	31	100	0.0	0.0	40	20	12.5	67.5
AMK	100	0	0	100	50	10	2	88	37	18.9	2.7	78.4	12	8.3	25	66.7	11	90.9	0.0	9.1	83	16.9	2.4	80.7
GEN	126	40	0.8	59.5	59	54	0.0	88	44	11.4	4.5	84.1	13	7.7	53.8	38.5	59	57.6	8.5	33.9	97	20.6	3.1	76.3
CIP	125	66	2.4	31.2	59	37	22	40.7	42	23.8	0.0	76.2	13	38.5	15.4	46.2	58	67.2	0.0	32.8	97	24.7	6.2	69.1
LVX	23	87	0.0	13	ND	ND	ND	ND	ND	ND	ND	ND	ND	ND	ND	ND	11	100	0.0	0.0	13	30.8	7.7	61.5
TGC	16	0.0	0.0	100	ND	ND	ND	ND	ND	ND	ND	ND	ND	ND	ND	ND	ND	ND	ND	ND	ND	ND	ND	ND
SXT	95	60	0.0	40	45	78	0	18	36	27.8	0.0	72.2	11	72.7	0	27.3	40	52.5	0.0	47.5	ND	ND	ND	ND

**Notes.**

AMP ampicillin FOX cefoxitin CXM cefuroxime CTX cefotaxime CAZ ceftazidime FEP cefepime MEM meropenem IPM imipenem SAM ampicillin/sulbactam TZP piperacillin/tazobactam AMK amikacin GEN gentamicin CIP ciprofloxacin LVX levofloxacin TGC tigecycline SXT trimethoprim-sulfamethoxazole ND not determined

**Table 6:** Distribution of resistant (R), intermediate (I), and susceptible (S) gram-positive isolates from pleural fluid of patients in Mexico.

	*S. aureus*				*E. faecalis*				*E. faecium*			
Antibiotic	n	%R	%I	%S	n	%R	%I	%S	n	%R	%I	%S
PEN	18	94.4	0.0	5.6	17	5.9	0.0	94.1	ND	ND	ND	ND
AMP	14	92.9	0.0	7.1	60	0	0.0	100	29	86.2	0	13.8
OXA	105	31.4	0.0	68.6	ND	ND	ND	ND	ND	ND	ND	ND
FOX	75	34.7	0.0	65.3	13	100	0.0	0.0	ND	ND	ND	ND
CIP	105	30.5	1	68.6	59	37.3	6.8	55.9	29	51.7	27.6	20.7
LVX	88	31.8	0	68.2	44	34.1	0.0	65.9	24	45.8	4.2	50
ERY	105	37.1	1.9	61	59	57.6	32.2	10.2	29	96.6	3.4	0.0
CLI	105	34.3	0.0	65.7	15	100	0.0	0	ND	ND	ND	ND
TET	90	8.9	0.0	91.1	45	73.3	0.0	26.7	23	52.2	0.0	47.8
GEN	105	12.4	5.7	81.9	13	100	0.0	0.0	ND	ND	ND	ND
SXT	106	2.8	0.0	97.2	14	92.9	0.0	7.1	ND	ND	ND	ND
RIF	83	2.4	0.0	97.6	ND	ND	ND	ND	ND	ND	ND	ND
LZD	107	1.9	0.0	98.1	60	0.0	3.3	96.7	28	0.0	0.0	100
TGC	12	0	0.0	100	11	0.0	0.0	100	9	0.0	0.0	100
VAN	105	1.9	0.0	98.1	59	3.4	0.0	96.6	29	27.6	0.0	72.4
CHL	ND	ND	ND	ND	13	15.4	0.0	84.6	ND	ND	ND	ND

**Notes.**

PEN penicillin AMP ampicillin OXA oxacillin FOX cefoxitin CIP ciprofloxacin LVX levofloxacin ERY erythromycin CLI clindamycin TET tetracycline GEN gentamicin SXT trimethoprim-sulfamethoxazole RIF rifampicin LZD linezolid TGC tigecycline VAN vancomycin CHL chloramphenicol ND not determined

## Discussion

In the present study, most bacterial species detected were gram-negative (when no *S. epidermidis* was included), with *E. coli* being the most frequent bacterial species detected in blood, CSF, and pleural fluid.

Regarding blood cultures, our results are similar to those reported in an Austrian study from 2006 to 2015 in which *E. coli* was the most common isolated pathogen (*n* = 2,869), followed by *S. aureus* (*n* = 1,439), *Enterococcus* spp. (*n* = 953), and *Klebsiella* spp. (*n* = 816) (Kreidl et al., 2019). Furthermore, a study in a seven-year North American study in which 24,179 cases of bloodstream infections were included, the most common organisms were coagulase-negative staphylococci (31% of isolates), followed by *S. aureus* (20%) and *Enterococci* (9%) (Wisplinghoff et al., 2004).

In our study, the most frequent species was *E. coli* (when no *S. epidermidis* was considered). It has been reported that this bacterial species is responsible for 48 per 100,000 person-years cases of bacteremia, increasing to 100 per 100,000 person-years in the 55–75 age group and over 300 per 100,000 person-years in the 75–85 age group (Bonten et al., 2021). These data underline the relevance of this bacterial species.

Commercial products dominate antimicrobial susceptibility testing methods in hospital laboratories, with relatively high accuracy. These methods have become better suited to fastidious species like *S*. *pneumoniae,* for which the need for red blood cells complicates the use of BMD. Compared with the BMD method, the proportion of penicillin-resistant *S. pneumoniae* was reported more frequently when routine, conventional methods were used. This was not observed for levofloxacin, erythromycin, trimethoprim-sulfamethoxazole, and vancomycin. A slight decrease in penicillin and cefotaxime resistance was observed in this study period compared to the previous decade, 2009–2018 (Garza-González et al., 2020), which might explain the continued use of pneumococcal conjugate vaccines since 2008. An increase in erythromycin resistance was observed, and a further increase is likely to be expected shortly, considering the use of azithromycin as an inflammatory aid in COVID-19 patients.

In blood cultures, the possibility of contamination must always be considered in interpreting positive results because microbes that are not in the bloodstream may be introduced into the bottle during blood collection (Gonsalves et al., 2009). Contamination is often due to organisms that can be part of the skin microbiota, such as *S. epidermidis*. In our study, the most frequent species detected was *S. epidermidis,* and according to our results, contamination seems excessive and points to the need for improving blood intake procedures. However, other pathogens detected in this study are not frequently observed in the skin (*e.g.*, *E. coli*, *K. pneumoniae. E. cloacae, S. marcescens*, *K. aerogenes*, and *P. mirabilis*). Thus, they are most probably from the clinical specimen. A small sample volume is associated with difficulties in maintaining sterile conditions due to poor venous access (Gonsalves et al., 2009; Bekeris et al., 2005); thus, the contamination rate inversely correlates with blood volume (Gonsalves et al., 2009; Bekeris et al., 2005). Additionally, venipuncture, arterial access, or central venous access are associated with different contamination rates: 36%, 10%, and 7%, respectively (Gonsalves et al., 2009). Our study has no such data to verify these values, but laboratories followed the manufacturer’s recommendations regarding volume in general.

In this study, the drug resistance of pathogens from blood was similar to our previous reports (Garza-González et al., 2021; Garza-González et al., 2020; Garza-González et al., 2019). This study added data on drug susceptibility for *Citrobacter freundii*, *Serratia marcescens*, *Proteus mirabilis*, *Morganella morganii*, and *Burkholderia cepacia*, for which limited information is available in Mexico.

Regarding *S.* Typhi, the widespread use of fluoroquinolones has led to the emergence of decreased ciprofloxacin susceptibility. Our previous report, with only 10 clinical isolates included, showed 20% resistance (Garza-González et al., 2019). This work detected an 11% resistance (with 88 clinical isolates included).

Regarding the *B. cepacia*, trimethoprim-sulfamethoxazole remains a recommended first-line therapy (Avgeri et al., 2009). Our report detected 20.5% resistance for this drug; another first-line therapy, meropenem, showed a resistance of 5%. The primary alternative therapeutic agents beyond trimethoprim-sulfamethoxazole include ceftazidime and meropenem (Avgeri et al., 2009). This study showed that the high susceptibility to ceftazidime (0% resistance, at least *in vitro*) renders this antibiotic a good alternative in the studied populations.

The bacterial pathogens detected in CSF are various according to age. Between 16 and 50 years, *Neisseria meningitidis* and *S. pneumoniae* are frequently reported. In those over 50 years, *Listeria monocytogenes* and aerobic gram-negative bacilli are encountered (van deBeek et al., 2016). Furthermore, in immunocompromised individuals over 50 years of age, aerobic gram-negative bacilli and *Salmonella* spp. are frequently found (van deBeek et al., 2016). Other gram-negative bacteria and *P. aeruginosa* are pathogens found after neurosurgery or head trauma (Tunkel et al., 2017). Regardless of age, the most frequent causative agents isolated from CSF have been reported to be gram-negative bacteria, with *A. baumannii, K. pneumoniae,* and *E. coli* as the most frequently recovered species (Hu et al., 2019). In our study, similar results were observed, with *E. coli* being the most prevalent, if we omitted *S. epidermidis*.

*S. epidermidis* may have relevance in patients with hydrocephalus controlled by diverting or shunting the fluid around the obstruction and into a suitable body cavity. Shunts may be colonized primarily by skin organisms, with *S. epidermidis* being the most common cause of shunt colonization (Yakut et al., 2018; Bayston, 1989). In our population, *S. epidermidis* was the most frequent bacterial species detected. Whether this organism was associated with shunt infections needs further exploration.

In China, 244,843 strains from 44 teaching hospitals were analyzed in 2018 (14.8% from blood and 1.3% from CSF). In CSF only, 64.1% of *K. pneumoniae* and 60% of *P. aeruginosa* were resistant to carbapenem (29). Fortunately, we detected much lower percentages of resistance (4.9% and 31.3%). These data support the need to monitor drug resistance in specific populations.

Aerobic organisms are most implicated in pleural infection. When all populations are considered, streptococcal species are the primary cause at approximately 60%, followed by staphylococcal species and *Enterococcus* (Davies et al., 2010). The gram-negative aerobes cause about 15% and anaerobes nearly 14% of identified pathogens (Davies et al., 2010).

When causative agents are stratified, causative agents vary considerably by the site of acquisition, age, or comorbidities. For example, 50% of cases of community-acquired empyema are due to streptococci, and the remainder is due to staphylococci, anaerobes, and gram-negative bacilli (Maskell et al., 2005). However, in hospital-acquired empyema (associated with hospital-acquired pneumonia) or iatrogenic causes, staphylococci, gram-negative bacilli, *Enterococcus* species, and cefoxitin-resistant *S. aureus* are the most frequently implicated organisms. In pediatric patients, the most frequent isolate in pleural fluids reported is *S. pneumoniae* (up to 48%), followed by group A *Streptococcus*, *S. aureus.* In patients with comorbidities, especially diabetes or alcoholism, gram-negative bacteria are the most frequent (Maskell et al., 2005). In our study, *E. coli* and *S. aureus* were most frequently detected, with a significant role of *P. aeruginosa* as the causative agent. Unfortunately, we have no data on the comorbidities associated with these infections.

The selection of appropriate empiric therapy results in better outcomes in severe infections occurring in sterile fluids, *i.e.,* blood, cerebrospinal and pleural fluids. The prevalent, most frequently isolated antibiotic-resistant pathogens in every hospital should guide the initial therapy selection. Also helpful is the knowledge of previous experiences of discordant therapy in bacteremia caused by frequent resistant pathogens like *S. aureus* or Enterobacteriaceae. Adjustments should be made according to the report on cultures and susceptibility (Kadri et al., 2018; Kadri et al., 2021). The results from this and other studies from the INVIFAR network will help in selecting the appropriate empiric therapy.

Part of the isolates was reported in a previous manuscript of the network, specifically, those collected from the second semester of 2019 and the second semester of 2020 (López-Jácome et al., 2021), exclusively from the centers that participated in both studies and from blood isolates.

In this study, each laboratory identified the strains and tested their susceptibilities using routine, conventional methods, including commercial microdilution systems. Each system has advantages and limitations, and the results vary widely by antimicrobial drugs, software versions, and cards used (Gajic et al., 2022).

Some studies have evaluated the performance of these instruments. For example, a multicenter evaluation showed that categorical agreement between the Phoenix system and the broth microdilution method for 2013 streptococcal isolates, including *S. pneumoniae*, ranged from 92% to 100% (Richter et al., 2007). Furthermore, the performance of the Phoenix, Vitek 2, and the MicroScan have been compared with the microdilution broth reference method for 311 clinical isolates of *S. pneumoniae,* and the overall essential agreement between each test was >95% (Mittman et al., 2009). Additionally, 109 *S. maltophilia* bloodstream isolates were analyzed, and very major errors were >.3% for trimethoprim-sulfamethoxazole (MicroScan, Phoenix), levofloxacin (MicroScan), and ceftazidime (all systems).

Limitations of this retrospective study are worth noting. First, we were unable to collect complete data on the age of the patients. Second, no clinical data were available to support the indication of the study or the relevance of the clinical isolate. Third, our results represent the combined data of general, mother and child, specialty hospitals, and other centers; thus, each hospital needs to address its results. Fourth, not all antibiotics were available from all centers; thus, some antibiotics are not included, such as levofloxacin for *P. aeruginosa.* Fifth, different methodologies were used for the detection of antibiotic susceptibility.

## Conclusions

The results presented in this article represent the consolidated analysis of isolates from 45 centers. *S. epidermidis* was the most frequently recovered bacterial species from blood and CSF. Whether these isolates represent contamination or are associated with infection remains unclear. Gram-negative bacteria, with *E. coli* most prevalent, are frequently recovered from CSF, blood, and pleural fluid. In *S. pneumoniae*, the routine, conventional methods can help detect resistance percentages for levofloxacin, cefotaxime, and vancomycin.

## Additional Information and Declarations

### Data Availability

The following information was supplied regarding data availability: The raw data, including the number of isolates analyzed, percentage of resistant, intermediate, and susceptible isolates included for each specimen, and bacterial species are available in Tables 1–6.
